# Impacts of 360 mg/kg Niacinamide Supplementation in Low-Protein Diets on Energy and Nitrogen Metabolism and Intestinal Microbiota in Growing–Finishing Pigs

**DOI:** 10.3390/ani15142088

**Published:** 2025-07-15

**Authors:** Xiaoyi Long, Haiyang Wei, Zhenyang Wang, Zhiru Tang, Yetong Xu, Xie Peng, Zhihong Sun, Liuting Wu

**Affiliations:** 1 Research Center for Bio-Feed and Molecular Nutrition, College of Animal Science and Technology, Southwest University, Chongqing 400715, China; 2 State Key Laboratory of Swine and Poultry Breeding Industry, College of Animal Science and Technology, Sichuan Agricultural University, Chengdu 611130, China

**Keywords:** growing–finishing pigs, niacinamide, microbial metabolome, intestinal function, nutrient metabolism

## Abstract

This study aimed to investigate the effects of adding 360 mg/kg niacinamide (NAM) to diets on nutrient metabolism, providing insights into how dietary NAM supplementation enhances nitrogen utilization and growth performance in pigs. Forty growing–finishing pigs were randomly assigned to one of four experimental diets as follows: basal diet + 30 mg/kg NAM (CON), basal diet + 360 mg/kg NAM (CON + NAM), low-protein diet + 30 mg/kg NAM (LP), and low-protein diet + 360 mg/kg NAM (LP + NAM). Results showed that supplementation of both the CON and LP diets with 360 mg/kg NAM resulted in decreased urea nitrogen concentrations and carbamyl phosphate synthetase-I activity (*p* < 0.05). The pyruvate dehydrogenase activity in the serum and liver, as well as the activity of pyruvate dehydrogenase, citrate synthase, and glutamate dehydrogenase 1 in the ileum mucosa, was increased by supplementing the LP diet with 360 mg/kg NAM (*p* < 0.05). The LP diet with 360 mg/kg NAM increased the villi length to crypt depth, mRNA expression of glucose transporters 1 and 2 and alanine-serine-cysteine transporter 1, and mRNA expression of mechanistic target of the rapamycin 1 in the ileum (*p* < 0.05). Additionally, 360 mg/kg NAM supplementation in the LP diet reduced ileal *Lactobacillus* abundance (LDA > 4) and increased ileal microbial nucleotide and purine metabolism (*p* < 0.05). Our findings suggest that addition of 360 mg/kg NAM to the LP diet reduced urea production in the liver, enhanced glucose and amino acid absorption and transport in the ileum, and improved glucose metabolism.

## Simple Summary

Enhancing nutrient metabolism, particularly amino acid metabolism, in animals is a crucial strategy for improving protein utilization efficiency, reducing nitrogen excretion, and conserving protein feed resources. In this study, we found that the use of high levels of nicotinamide effectively reduced urea nitrogen production in the liver of growing–finishing pigs, enhanced glucose and amino acid absorption in the ileum, and altered microbial nucleotide and purine metabolism. These metabolic alterations were accompanied by improved intestinal morphology and elevated activity of key tricarboxylic acid cycle enzymes in the ileum. These findings not only enhance our understanding of the role of dietary nicotinamide in modulating host–microbiome interactions but also establish a theoretical foundation with minimized nitrogen excretion.

## 1. Introduction

Diets with protein levels exceeding the nutritional requirements of animals reduce nutrient metabolism efficiency and increase nitrogen excretion, leading to resource waste and environmental pollution [1]. Low-protein (LP) diets are the most effective strategy to address nitrogen excretion pollution in livestock [2,3,4]. However, lowering the crude protein (CP) content in animal feed requires supplementing essential amino acids (AAs) to maintain a balanced dietary AA profile [5]. Furthermore, the consumption of LP diets has been associated with fat accumulation during the growth-finishing phases in pigs [6], which limits the widespread use of LP diets in livestock husbandry.

Nicotinamide adenine dinucleotide (NAD^+^), a molecule present in nearly all mammalian cells, is essential for cellular redox balance and basal energy metabolism [7,8]. Extensive research has explored the use of NAD^+^ in mammalian tissues [9]. The silent information regulator sirtuin 1 (SIRT1) interacts with adenosine monophosphate (AMP) protein kinases via NAD^+^ to regulate nutrient metabolism [10]. Additionally, NAD^+^ modulates the mechanistic target of the rapamycin 1 (mTOR1) pathway, playing a pivotal role in protein metabolism [11]. Therefore, modulating NAD^+^ metabolism to enhance glucose oxidative energy delivery, thereby reducing the reliance on AA as an energy source, presents a promising strategy for improving nitrogen conservation.

The salvage pathway, an essential NAD^+^ synthesis route in mammals, generates NAD^+^ from nicotinamide (NAM) [12,13], which is vital for NAD^+^ production [14,15]. Microbial NAM enzymes play a crucial role in the transformation of NAM into nicotinic acid, a process vital for metabolic protection [16]. The gut microbiome transforms orally administered NAM riboside, a common nutraceutical, into nicotinic acid, contributing to host NAD^+^ metabolism [17]. Furthermore, NAM nucleoside supplements and oral NAM enhance bacterial-mediated deamidation, promoting NAD^+^ synthesis in several tissues [16]. Therefore, investigating the gut microbiome and associated metabolites, which are crucial for host NAD^+^ metabolism, is essential.

In our previous study, supplementing the control (CON) and LP diets with 360 mg/kg of NAM significantly reduced the nitrogen emissions and improved the growth performance of pigs [18]; however, the mechanistic basis underlying dietary nicotinamide’s modulation of nitrogen metabolism remains poorly elucidated. This study aimed to investigate the effects of high dietary NAM supplementation on intestinal and liver metabolism and gut microbiota, elucidating its role in improving the nitrogen conservation and growth outcomes of growing–finishing pigs.

## 2. Materials and Methods

### 2.1. Animal Use, Care, and Ethical Considerations

Forty pigs (Duroc × Landrace × Large White; body weight 37 ± 1.0 kg; age 70 days) were used in the trial. Stainless steel cages (1.8 m in length × 1.2 m in height × 1.0 m in width) were used to accommodate the pigs, and each pig was individually housed in a cage. The cages were equipped with water nipples, ensuring ad libitum access to water. Thermostatically controlled heaters and exhaust blowers were used to maintain the room temperature at 21.0–24.0 °C.

All experimental procedures were approved by the Southwest University Animal Care and Use Committee (Ethical license number: IACUC-20210530-02) on 30 May 2021.

### 2.2. Diets and Experimental Design

Four experimental diets were established: (1) basal diet (for two periods, namely the growing diet and the finishing diet, which are included in Tables S1 and S2) supplemented with 30 mg/kg NAM (CON), (2) basal diet supplemented with 360 mg/kg NAM (CON + NAM), (3) LP diet supplemented with 30 mg/kg NAM (LP), and (4) LP diet supplemented with 360 mg/kg NAM (LP + NAM). The trial lasted 11 weeks, including a one-week acclimation phase for the CON diet. The CON diet was formulated to meet the recommendations of the National Research Council [19]. Lysine, methionine, tryptophan, and threonine were integrated into all trial diets, except for the CON diet, to ensure that the AA levels were as high as those of the CON diet. The pigs in the four groups received a feeding restriction diet of 45 g/kg body weight. Each pig’s daily feed was evenly distributed between two meals, which were served at 7:00 a.m. and 5:00 p.m. The experimental diet components and nutrient contents are presented in Supplementary Tables S1 and S2.

### 2.3. Recording and Sample Collection

The daily feed intake of each group was recorded. The weights of the pigs were documented at the beginning and the conclusion of the trial. After finishing the trial, six pigs in each group with the closest weight to the average group weight were slaughtered. Before slaughter, a 5 mL blood sample was obtained via jugular venipuncture and transferred into a 10 mL tube that had been pretreated with sodium heparin for anticoagulation. After 2 h of incubation at room temperature (~25 °C), the blood samples were centrifuged at 3000 rpm for 20 min. The supernatant was collected and stored at −80 °C. Pentobarbital sodium at a dose of 50 mg/kg body weight was injected into the jugular vein of the pig for anesthesia. The jugular vein and carotid artery were severed to exsanguinate the pigs. After opening the abdominal cavity, the right lobe of the liver was collected using surgical shears, cleaned with sterile saline, and wrapped in sterile foil to prevent contamination. A 30 cm segment of the mid-ileum was ligated with surgical sutures to collect digesta from the ileum. The segment was removed from the abdominal cavity after the mesentery was separated. One end was carefully opened to gather an appropriate volume of digesta into a 2 mL sterile cryotube for storage. After rinsing once with regular saline, the middle segment of the ileal mucosa (scraped with aseptic slides) was wrapped in aseptic tin foil. The digesta, ileum mucosa, and liver samples were promptly cryopreserved in liquid nitrogen and then kept at −80 °C for analysis.

### 2.4. Chemical Analysis and Calculation

The dietary compositions, encompassing the dry matter content, overall feed composition, and the calcium and phosphorus levels, were analyzed as previously described [20].

For serum metabolites and hormones, kits (Jiancheng Biotechnology Research Institute, Nanjing, China) were used to measure blood urea nitrogen (BUN, C013-2-1), glucose (A154-1-1), low-density lipoprotein cholesterol (A113-1-1), high-density lipoprotein cholesterol (A112-1-1), triglycerides (A110-1-1), growth hormone (H091-1-1), fasting insulin (H203-1-1), and glycogen (A043-1-1) concentrations, as well as glutamic-pyruvic transaminase (C009-2-1), glutamic-oxaloacetic transaminase (C010-2-1), pyruvate dehydrogenase (PDH, H262-1-1), and lactate dehydrogenase (LDH, A020-2-2) activities in serum. The homeostatic model assessment for HOMA-IR was determined by multiplying the fasting insulin level (mIU/L) by the fasting blood glucose level (mmol/L) and dividing the result by 22.5 [21].

For the activities of enzymes participating in the urea cycle, the total protein (A045-4-2) and urea nitrogen (C013-2-1), glutamic-oxaloacetic transaminase (C010-2-1), glutamic-pyruvic transaminase (C009-2-1), CPS-1 (H557-1), ornithine transcarbamylase (H292), glutamine synthetase (A047-1-1), and glutaminase (A124-1-1) activities were assessed using assay kits (Jiancheng Biotechnology Research Institute, Nanjing, China).

Of the activities of enzymes involved in nutritional metabolism, the enzymatic activity levels of citrate synthase (CS, G0834F) and glutamate dehydrogenase 1 (GDH1, G0405W) in the liver and ileum of pigs were analyzed using kits from Grace Biotechnology (Suzhou, Jiangsu, China). The carnitine palmitoyltransferase 1 (H681-1-1), PDH (H262-1-1), LDH (A020-2-2), and hexokinase (HK, A077-4-1) activities in the liver and ileum of pigs were analyzed using assay kits (Nanjing Jiancheng Biotechnology Research Institute, Nanjing, China).

Regarding ileal mucosa morphology, ileal samples were preserved in 4% formalin. The general histological examination was performed using hematoxylin and eosin staining [22].

The mRNA expression of genes related to nutrient metabolism was determined using fluorescent real-time quantitative PCR. First, liver and ileum tissues, which included an RNA preservation solution to prevent RNA degradation, were processed using a high-throughput tissue grinder to achieve a fine grind (Scientz Biotechnology, Ningbo, China). Total RNA was extracted from liver and ileum tissue using the SteadyPure RNA Extraction Kit (Accurate Biology, Changsha, China). The extracted RNA samples were tested for degradation using 1.0% agarose gel electrophoresis. cDNA was synthesized by aspirating 1000 ng of RNA using a commercial reverse transcription kit according to the manufacturer’s instructions (Applied Biological Materials Biotechnology, Vancouver, BC, Canada). Finally, fluorescence real-time quantitative PCR was performed using the Bio-Rad CFX Connect Real-Time System (Bio-Rad, Hercules, CA, USA). The fluorescence real-time quantitative PCR reaction mix comprised 5.0 μL of SYBR Green Supermix (2×), 0.4 μL of each upstream and downstream primer, 0.4 μL of downstream primer, 2 μL of cDNA, and 2.2 μL of ddH2O. The primers were designed according to the relevant gene sequences in NCBI GenBank (pig species), tested for primer specificity using BLAST (https://www.ncbi.nlm.nih.gov/, accessed on 10 April 2023) and synthesized by Shanghai Sangong Bioengineering Co. (Shanghai, China) (Table 1). The PCR cycle initiated with 3 min at 95 °C, followed by 41 cycles for 10 s at 95 °C, 30 s at 58 °C for *NAMPT* and *ACC1*, 59 °C for *SIRT1*, liver kinase B1, *AMPK1*, peroxisome proliferator-activated receptor (*PPAR*), *FOXO1*, cAMP response element-binding (*CREB*), *GLUT1*, *GLUT2*, *ASCT1*, and *ASCT2*, and 58 °C for peroxisome proliferator-activated receptor coactivator 1 (*PGC-1*), *mTORC1*, and *SLC7A5*, and 30 s at 72 °C. The amplification specificity was checked by observing the melting curves. All PCR amplification efficiencies were 90–110%. The relative expression of the target genes was analyzed using the 2^–∆∆Ct^ method, which was calculated based on the normalization approach.

Assessing ileal microbiota, ileal digesta that had been preserved were utilized to investigate the diversity of intestinal bacteria in the colon through 16S rRNA gene sequencing on the Illumina Novaseq sequencing platform. The assay procedure was carried out as previously described [23,24,25].

For ileal microbial metabolomics, aqueous and organic metabolites were extracted as previously described [26]. The raw liquid chromatography–mass spectrometry data were processed using Software Analyst 1.6.3 and MultiQuant 3.0.3. Chromatographic peaks corresponding to the analytes in various samples were subjected to integration correction using reference retention times and peak shape information obtained from standard samples. These steps were implemented to ensure the precision and reliability of both qualitative and quantitative analyses. The data were subjected to quality control using TIC, PCA, Cluster, and RSD. The metabolites were identified and annotated using the MWDB database 4.4. Fold change analysis and orthogonal partial least squares discriminant analysis were employed to screen for differential metabolites. Metabolites with variable influence on projection (VIP) ≥ 1, fold change ≥ 2, or fold change ≤ 0.5 were selected. Differences in metabolite levels for each comparison were visually represented using VIP, *p*-value, and fold change. The Kyoto Encyclopedia of Genes and Genomes (KEGG, http://www.genome.jp/kegg, accessed on 3 May 2023) database was used to perform pathway enrichment analysis of the differential metabolites.

For association analysis between the ileal microbiota and metabolome, operational Taxonomic Units (OTUs) obtained from analyzing ileal microbial diversity can elucidate the relationship between metabolites and OTUs, as well as the metabolic groups of ileal microbes, enabling further analysis of population structure, physiological metabolism, and genetic variation in microorganisms. Before the analysis, all OTUs were normalized by dividing the expression level of each OTU by the sum of the expression levels of all OTUs. The association analysis between the ileal microbiome and microbial metabolome was examined using Spearman’s correlation test, with *p*-values of <0.01 deemed extremely significant and <0.05 regarded as significant. The correlation results between metabolites and OTUs were assessed based on the correlation coefficient *p*-value criterion: |r| ≥ 0.6, *p*-value < 0.05. A correlation heat map was created using the “Python 3.7” module of the R programming language 4.2 (https://www.bioinformatics.com.cn, accessed on 5 June 2023).

### 2.5. Statistical Analysis

SAS 8.1 and GraphPad Prism version 8.0 (GraphPad Software, La Jolla, CA, USA) were used to conduct statistical analyses. A two-way analysis of variance (ANOVA) was used to evaluate the effects of dietary CP concentration, NAM concentration, and the interaction between the two. The statistical significance of the disparities observed between the various treatments was determined using Tukey’s multiple comparison test. A *p*-value of <0.05 was considered significant.

## 3. Results

### 3.1. Serum Biochemical Parameters

The serum biochemical parameters of the growing and finishing pigs are presented in Table 2. Pigs fed the CON diet exhibited significantly higher serum blood urea nitrogen (BUN) and growth hormone (GH) concentrations than those fed the LP diet (*p* < 0.05). Supplementing pig diets with 360 mg/kg NAM increased glucose (GLU) concentration (*p* < 0.05) and pyruvate dehydrogenase (PDH) activity (*p* < 0.05) in the serum compared to 30 mg/kg NAM. Pigs fed the low-protein diet exhibited significantly lower serum PDH activity (*p* < 0.05) than pigs fed the normal-protein diet. In pigs fed the CON + NAM diet, a higher high-density lipoprotein cholesterol (HDL-C) concentration was observed than in both pigs fed the CON diet (*p* < 0.05) and the LP + NAM diet (*p* < 0.05). Insulin resistance (HOMA-IR) values were significantly higher (*p* < 0.05) in pigs fed 360 mg/kg NAM compared to those fed 30 mg/kg NAM.

### 3.2. Liver Urea Cycle-Related Indicators

The liver urea cycle-related indicators for all groups are presented in Table 3. Liver carbamyl phosphate synthetase-I (CPS-1) activity was significantly higher in pigs fed a diet supplemented with 30 mg/kg NAM than in those fed a diet supplemented with 360 mg/kg NAM (*p* < 0.05). The liver glutamine synthetase (GS) activity in pigs fed the low-protein diet was significantly lower than in those fed the normal-protein diet (*p* < 0.05).

### 3.3. Limiting Enzymes in Liver and Ileal Mucosa Related to Nutrient Metabolism

The activities of the liver and ileal mucosa tricarboxylic acid (TCA) cycle-related enzymes are presented in Table 4. Pigs fed the low-protein diet exhibited significantly lower liver glutamate dehydrogenase 1 (GDH1) activity (*p* < 0.05) than pigs fed the normal-protein diet. Carnitine palmitoyltransferase 1 (CPT1) activity was significantly lower (*p* < 0.05) in the liver of pigs fed the diet supplemented with 360 mg/kg NAM than in those fed the diet supplemented with 30 mg/kg NAM. Supplementing pig diet with 360 mg/kg NAM increased liver PDH activity (*p* < 0.05) compared to 30 mg/kg NAM. Liver hexokinase (HK) activity was significantly higher in pigs fed the low-protein diet than in those fed the normal-protein diet (*p* < 0.05). In addition, HK activity significantly lower in the liver of pigs fed the 30 mg/kg NAM diet than in those fed the 360 mg/kg NAM diet (*p* < 0.05). Moreover, pigs treated with the CON + NAM diet increased citrate synthase (CS) compared to those treated with the CON, LP, and LP + NAM diets in the ileal mucosa (*p* < 0.05). The CON diet increased citrate synthase (CS) in the liver of pigs compared to the LP diet (*p* < 0.05). The GDH1 activity was significantly lower in the liver of pigs fed the low-protein diet than in those fed the normal-protein diet (*p* < 0.05), while the GDH1 activity was significantly higher in the ileal mucosa of pigs fed the 360 mg/kg NAM diet than in those fed the 30 mg/kg NAM diet (*p* < 0.05). Pigs fed the low-protein diet exhibited significantly higher CPT1 activity (*p* < 0.05) than pigs fed the normal-protein diet in the ileal mucosa. Ileal mucosal PDH activity was significantly lower in pigs fed the low-protein diet than in those fed the normal-protein diet (*p* < 0.05). The PDH activity in the ileal mucosa was higher in pigs fed the diet supplemented with 30 mg/kg NAM than in those fed the diet supplemented with 360 mg/kg NAM (*p* < 0.05).

### 3.4. Ileal Mucosa Morphology

**Figure 1:**
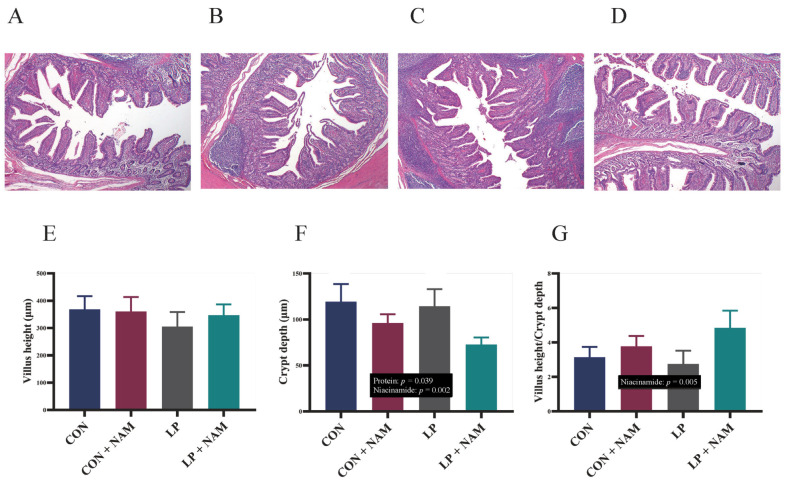
Effects of dietary CP and niacinamide contents on the ileum morphology of pigs (HE staining; magnification: 40×). (**A**–**D**) represent CON, CON + NAM, LP, and LP + NAM groups; (**E**) the villus height; (**F**) the crypt depth; and (**G**) the ratio of villus height and crypt depth. Dietary treatments: CON, basal diet + 30 mg/kg NAM; CON + NAM, basal diet + 360 mg/kg NAM; LP, low-protein diet + 30 mg/kg NAM; LP + NAM, low-protein diet + 360 mg/kg NAM. Data are shown as mean ± SEM (*n* = 6).

A general histological analysis of the ileum is depicted in Figure 1 (Figure 1A–D). Crypt depth was significantly shallower in pigs fed the low-protein diet versus the normal-protein diet (*p* < 0.05) and in those fed 360 mg/kg NAM versus 30 mg/kg NAM (*p* < 0.05). The ratio of villus height to crypt depth in the ileum was significantly higher in pigs fed the 360 mg/kg NAM diet than in those fed the 30 mg/kg NAM diet (*p* < 0.05; Figure 1F,G).

### 3.5. mRNA Expression of Molecules in the Ileum Mucosa Involved in Glucose and Glutamine Absorption and Transport

**Figure 2:**
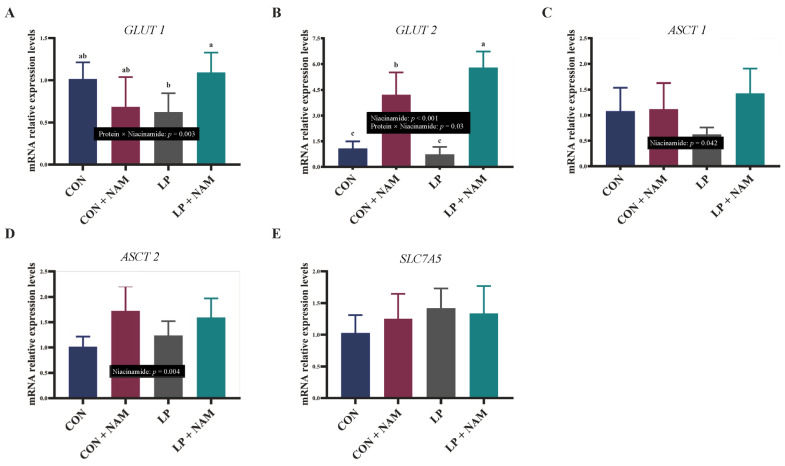
Effects of dietary CP and niacinamide on the mRNA expression of genes related to glucose and glutamine absorption and transport in the ileum of growing–finishing pigs. (**A**) *GLUT1*, glucose transporter 1; (**B**) *GLUT2*, glucose transporter 4; (**C**) *ASCT1*, glanine-serine-cysteine transporter 1; (**D**) *ASCT2*, glanine-serine-cysteine transporter 2; (**E**) *SLC7A5*, solute carrier family 7 member 5. ^a–c^ Values within a row with different superscripts differ significantly (*p* < 0.05). Dietary treatments: CON, basal diet + 30 mg/kg NAM; CON + NAM, basal diet + 360 mg/kg NAM; LP, low-protein diet + 30 mg/kg NAM; LP + NAM, low-protein diet + 360 mg/kg NAM. Data are shown as the mean ± SEM (*n* = 5).

An increase in mRNA expression was observed for glucose transporter 1 (*GLUT1*) in the ileal mucosa of pigs fed the LP + NAM diet compared with those fed the LP diet (*p* < 0.05; Figure 2A). Significantly higher *GLUT2* mRNA expression was observed in the ileal mucosa of pigs fed CON + NAM and LP + NAM diets than in those feds with CON and LP diets (*p* < 0.05; Figure 2B). In addition, the pigs in the LP + NAM group showed higher ileum mucosa *GLUT2* mRNA expression than pigs in the CON + NAM group. Alanine-serine-cysteine transporter 1 (*ASCT1*) mRNA expression in the ileum mucosa was significantly higher in pigs fed the 360 mg/kg NAM diet than those fed the 30 mg/kg NAM diet (*p* < 0.05; Figure 2C). Pigs fed the diet with 360 mg/kg NAM exhibited a higher *ASCT2* mRNA expression level in the ileal mucosa than those fed the diet with 30 mg/kg NAM (*p* < 0.05; Figure 2D). No significant difference was observed in the ileal mucosal mRNA expression of solute carrier family 7 member 5 (*SLC7A5*) among the four groups (*p* > 0.05; Figure 2E).

### 3.6. mRNA Expression of Molecules in the Liver Involved in NAD^+^ Metabolism

**Figure 3:**
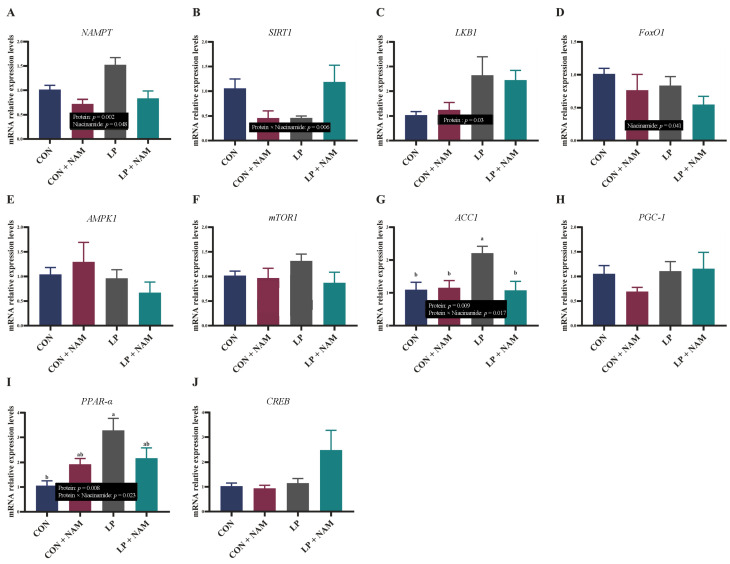
Effects of dietary CP and niacinamide content on the mRNA expression of genes related to the NAD^+^ signaling pathway in the liver of growing–finishing pigs. (**A**) Nicotinamide phosphoribosyltransferase (*NAMPT*). (**B**) Sirtuin-1 (*SIRT1*). (**C**) Liver kinase B1 (*LKB1*). (**D**) Forkhead box transcription factor O1 (*FOXO1*). (**E**) AMP-activated kinase alpha 1 (*AMPK1*). (**F**) The mechanistic target of rapamycin 1 (*mTOR1*). (**G**) Acetyl-CoA carboxylase 1 (*ACC1*). (**H**) Peroxisome proliferator-activated receptor coactivator 1 (*PGC-1*). (**I**) Peroxisome proliferator-activated receptor (*PPAR-α*). (**J**) cAMP response element-binding (*CREB*). ^a,b^ Values within a row with different superscripts differ significantly (*p* < 0.05). Dietary treatments: CON, basal diet + 30 mg/kg NAM; CON + NAM, basal diet + 360 mg/kg NAM; LP, low-protein diet + 30 mg/kg NAM; LP + NAM, low-protein diet + 360 mg/kg NAM. Data are shown as the mean ± SEM (*n* = 5).

The relative expression levels of genes involved in NAD^+^ metabolism in the liver are presented in Figure 3. Nicotinamide phosphoribosyltransferase (*NAMPT*) mRNA expression was significantly higher in the liver of pigs fed the low-protein diet compared with those fed the normal-protein diet, and liver *NAMPT* mRNA expression in the pigs fed the diet with 30 mg/kg NAM was higher than in those fed the diet with 360 mg/kg NAM (*p* < 0.05; Figure 3A). The interaction effect between CP and NAM on Sirtuin-1 (*SIRT1*) mRNA relative expression was statistically significant (*p* < 0.05; Figure 3B), but no significant differences were detected between the four groups (*p* > 0.05). Liver kinase B1 (*LKB1*) mRNA levels in pigs treated with the low-protein diet was significantly higher than in those treated with the normal-protein diet (*p* < 0.05; Figure 3C). The forkhead box transcription factor O1 (*FOXO1*) mRNA levels in the liver of pigs treated with 360 mg/kg NAM was significantly lower than in those treated with 30 mg/kg NAM (*p* < 0.05; Figure 3D). Significantly higher acetyl-CoA carboxylase 1 (*ACC1*) mRNA expression was observed in the liver of pigs fed the LP diet than in those fed with the CON, CON + NAM, and LP diets (*p* < 0.05; Figure 3G). Significantly higher peroxisome proliferator-activated receptor α mRNA expression was observed in the liver of pigs fed the LP diet compared to those fed the CON diet (*p* < 0.05; Figure 3I). There were no significant differences in the mRNA expression of AMP-activated kinase alpha 1 (*AMPK1*), mechanistic target of rapamycin 1 (*mTOR1*), peroxisome proliferator-activated receptor coactivator 1 (*PGC-1*), and cAMP response element-binding (*CREB*) in the liver of pigs among the four groups (*p* > 0.05; Figure 3E,F,H,J).

### 3.7. mRNA Expression of Genes in the Ileum Mucosa Involved in NAD^+^ Metabolism

**Figure 4:**
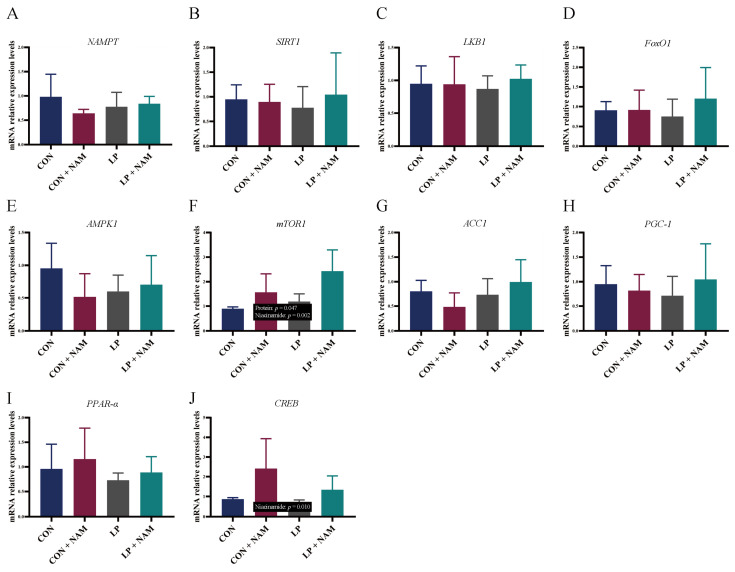
Effects of dietary CP and niacinamide content on the mRNA expression of genes related to the energy metabolism signaling pathway in the ileum of growing–finishing pigs. (**A**) Nicotinamide phosphoribosyltransferase (*NAMPT*). (**B**) Sirtuin-1 (*SIRT1*). (**C**) Liver kinase B1 (*LKB1*). (**D**) Forkhead box transcription factor O1 (*FOXO1*). (**E**) AMP-activated kinase alpha 1 (*AMPK1*). (**F**) The mechanistic target of rapamycin 1 (*mTOR1*). (**G**) Acetyl-CoA carboxylase 1 (*ACC1*). (**H**) Peroxisome proliferator-activated receptor coactivator 1 (*PGC-1*). (**I**) Peroxisome proliferator-activated receptor (*PPAR-α*). (**J**) cAMP response element-binding (*CREB*). Dietary treatments: CON, basal diet + 30 mg/kg NAM; CON + NAM, basal diet + 360 mg/kg NAM; LP, low-protein diet + 30 mg/kg NAM; LP + NAM, low-protein diet + 360 mg/kg NAM. Data are shown as the mean ± SEM (*n* = 5).

As Figure 4 shows, there were no differences in the mRNA expression of *NAMPT*, *SIRT1*, *LKB1*, *FOXO1*, *AMPK1*, *ACC1*, *PGC-1*, and *PPAR-α* in the ileum mucosa of pigs among the four groups (*p* > 0.05).

*mTOR1* mRNA expression in the ileum was significantly higher in pigs fed the 360 mg/kg NAM diets than in pigs fed the 30 mg/kg NAM diet; it was also significantly higher in pigs fed the low-protein diets than in pigs fed the normal-protein diet (*p* < 0.05; Figure 4F). Additionally, pigs fed the 360 mg/kg NAM diet had a significantly higher cAMP response element-binding protein mRNA expression level in the ileum than pigs fed the 30 mg/kg NAM diets (*p* < 0.05; Figure 4J).

### 3.8. Changes in the Ileum Microbiota

**Figure 5:**
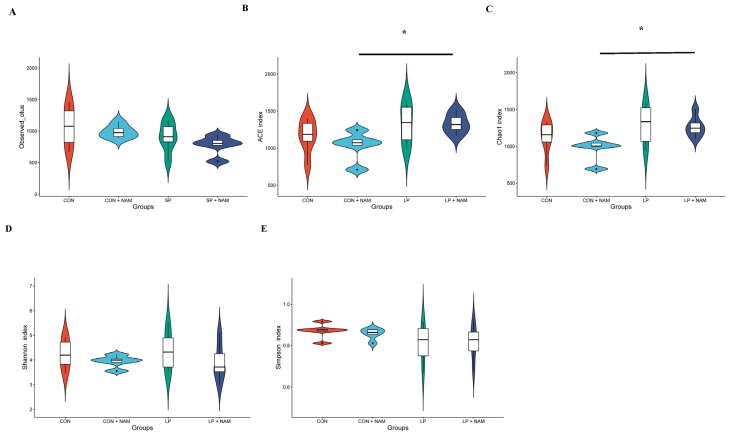

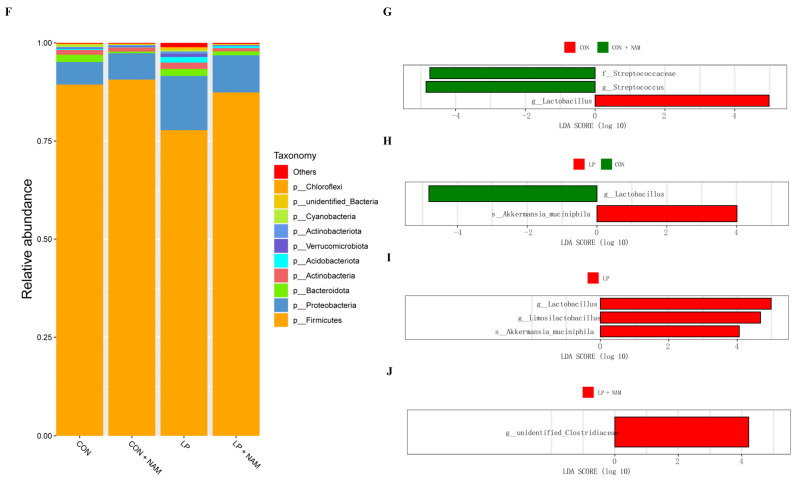
Effects of dietary CP and niacinamide on the alpha diversity of bacterial communities and microflora composition in the ileum of growing–finishing pigs (*n* = 6). (**A**–**E**) Alpha diversity was measured using the inverse. (**A**) Observed OTUs index. (**B**) ACE index. (**C**) Chao1 index. (**D**) Shannon index. (**E**) Simpson index. (**F**) Composition and relative abundance of bacterial phyla in different groups. * *p* < 0.05. (**G**–**J**) Histogram of the LDA value distribution. (**G**) CON vs. CON + NAM groups. (**H**) CON vs. LP groups. (**I**) LP vs. LP + NAM groups. (**J**) CON + NAM vs. LP + NAM groups. The histogram’s length signifies the impact of various species, while distinct colors denote different species groups. Linear discriminant analysis effect size (LEfSe) analysis was used to evaluate the significant differences, with a linear discriminant analysis (LDA) score of >4 and a *p* value of <0.05.

Figure 5 and Supplementary Figure S1 depict the ileal digesta microbiota of the pigs. The dilution curves for the samples (Supplementary Figure S1A) and the accumulation curve for relative abundance (Supplementary Figure S1B) indicate that the depth of a single sample was sufficient for the subsequent analysis. Alpha diversity indices, which assess the richness and diversity of bacterial communities within a sample, were calculated for the ileal digesta microbiota. The richness of the bacterial communities was assessed based on the observed OTUs, ACE, and Chao1 indices (Figure 5A–C), whereas diversity was estimated using the Shannon and Simpson indices (Figure 5D,E). The ACE and Chao1 indices of the pig ileal digesta microbiota CON + NAM group were significantly lower than those in the LP + NAM group (*p* < 0.05; Figure 5B,C).

At the phylum level, Firmicutes dominated the ileal microbiome of growing and finishing pigs. The predominant microbiota in all four groups were Proteobacteria, Bacteroidetes, and Actinobacteria (Figure 5F). *Lactobacillus* was differentially enriched among the gut bacterial communities in pigs fed diets without NAM supplementation compared to those fed 360 mg/kg NAM-supplemented diets (LDA score > 4; Figure 5G,I). Furthermore, bacteria from the Streptococcaceae family and the Streptococcus genus were differentially enriched in the gut bacterial communities of pigs fed the CON + NAM diet. Additionally, *Akkermansia muciniphila* was distinctively more abundant in the gut bacterial communities of pigs fed the LP diet than in those fed the CON diet (LDA score > 4; Figure 5G,H). Additionally, Lactobacilli were differentially enriched among the gut bacterial communities of pigs in the CON group compared to those in the LP group (LDA score > 4; Figure 5H). Compared to pigs fed the LP + NAM diet, the gut bacterial communities of pigs fed the LP diet exhibited a differential enrichment of bacteria from the *Lactobacillus*, *Limosilactobacillus*, and *A. muciniphila* species (LDA score > 4; Figure 5I). Compared to pigs in the CON + NAM group, the LP + NAM group showed differential enrichment of bacteria belonging to unidentified_Clostridiaceae among the gut bacterial communities (LDA score > 4; Figure 5J).

### 3.9. Metabolomic Analysis of Ileum Microbes

**Figure 6:**
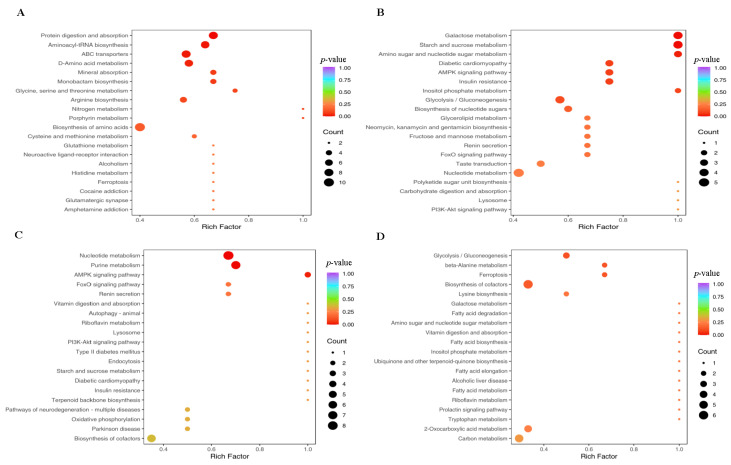
Effects of dietary CP and niacinamide content on ileal microbial metabolism in growing–finishing pigs (*n* = 6). (**A**–**D**) KEGG annotation classification and enrichment analysis of ileal microbes. (**A**) CON vs. CON + NAM group. (**B**) CON vs. LP groups. (**C**) LP vs. LP + NAM groups. (**D**) CON + NAM vs. LP + NAM groups. Each bubble on the KEGG map signifies a distinct metabolic pathway. This study involves KEGG annotation, categorization, and enrichment analysis of ileal digesta samples. The X-axis depicts the ratio of differential metabolites within each pathway to the total number of annotated differential metabolites in that pathway. The larger the bubble, the higher the ratio of differential metabolites. The Y-axis represents the metabolic pathway under enrichment analysis. The degree of redness in the bubble’s color is positively correlated with the level of enrichment of that pathway.

Differences in the metabolites of ileal microbes (Supplementary Table S3) among the four groups were determined using principal component analysis and orthogonal partial least-squares discriminant analysis. The KEGG classification pathway and enrichment analysis results of ileal digesta metabolites in growing and finishing pigs fed the experimental diets are presented in Figure 6. The differential metabolites in the ileal microbes of pigs in the four groups belonged to various metabolic pathways. Compared to the CON group, the CON + NAM group exhibited downregulated protein digestion and absorption, aminoacyl-tRNA biosynthesis, ABC transporter activity, and D-amino acid metabolism, specifically involving the metabolites L-aspartate, glutamine (Gln), threonine, L-glutamic acid, and serine (*p* < 0.05; Figure 6A). Additionally, in the ileal microbes of the LP group, starch and sucrose metabolism (metabolites: D(+)-glucose and trehalose-6-phosphate) and galactose metabolism (metabolite: D(+)-glucose) were significantly downregulated compared to those in the CON group (*p* < 0.05; Figure 6B). Compared with pigs fed the LP diet, pigs fed the LP + NAM diet exhibited upregulated nucleotide and purine metabolic pathways (metabolites: guanosine-diphosphate, dAMP, guanosine, adenine, and inosine) and the AMP-activated kinase alpha 1 (AMPK1) signaling pathway (metabolite: pyruvic acid) in ileal microbes (*p* < 0.05; Figure 6C). No significant differences were observed in the metabolic enrichment pathways when comparing the CON + NAM and LP + NAM groups (*p* > 0.05; Figure 6D).

### 3.10. Association Between the Ileum Microbiome and Metabolome

**Figure 7:**
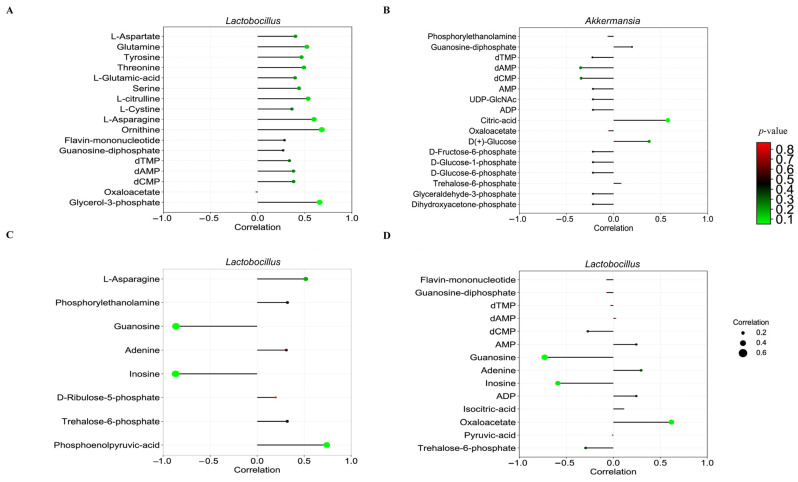
Correlation between ileum microorganisms and their differential metabolites in the ileum microbes (*n* = 6). (**A**) CON vs. CON + NAM group. (**B**) CON vs. LP groups. (**C**) LP vs. LP + NAM groups. (**D**) CON + NAM vs. LP + NAM group.

The correlation between each of the significant differential metabolites in the ileal digesta of pigs and *Lactobacillus* and *Akkermansia* in the ileal microbiota was determined using Pearson’s method (Supplementary Table S4). The correlation coefficient *p*-value was used as a screening condition to generate the correlation coefficient lollipop plot in Figure 7.

Compared to the CON group, the CON + NAM group exhibited a positive correlation between the ornithine (r = 0.68 and *p* < 0.05) and glycerol-3-phosphate (r = 0.66 and *p* < 0.05) concentrations in the ileal microbes and the *Lactobacillus* abundance (Figure 7A). In the LP group, a positive correlation was observed between the *Akkermansia* abundance and the citric acid concentration (r = 0.58 and *p* < 0.05) in the ileum of pigs compared to that in the CON group (Figure 7B). Compared with the CON group, the LP + NAM group exhibited a significant negative correlation in the ileum of pigs between the abundance of *Lactobacillus* and the inosine (r = –0.87 and *p* < 0.05) and guanosine (r = –0.86 and *p* < 0.05) concentrations in the ileal microbes (Figure 7C). Compared with the CON group, pigs in the LP + NAM group exhibited a significant positive in the ileum between the abundance of *Lactobacillus* and the concentration of phosphoenolpyruvic acid (r = 0.74 and *p* < 0.05). Furthermore, compared with the LP group, pigs in the LP + NAM group demonstrated a notable negative correlation in the ileum between *Lactobacillus* abundance and guanosine (r = –0.73 and *p* < 0.01) and inosine (r = –0.59 and *p* < 0.05) levels. Conversely, a significant positive correlation was observed in the ileum of pigs between oxaloacetate concentrations (r = 0.62 and *p* < 0.05) and *Lactobacillus* abundance (Figure 7D).

## 4. Discussion

In the present study, four different diet groups were used for feeding pigs to investigate whether NAM supplementation could be a metabolic regulator for nutrient metabolism in the liver and intestines in growing and finishing pigs fed with the LP diet, thereby increasing protein deposition and reducing AA wastage.

The body metabolizes excess AAs into ammonia to maintain nitrogen balance. Ammonia is converted to urea in the liver through the urea cycle and is excreted [27]. This study revealed that pigs fed the LP diet exhibited significantly lower serum BUN levels compared to those fed the CON diet. However, supplementation with 360 mg/kg NAM abolished this effect. This finding highlights the crucial role NAM plays in nitrogen preservation, indicated by the decreased liver CPS-1 activity. CPS-1 is an essential enzyme in the urea cycle, and its activity is associated with hepatic urea formation [28,29,30]. Therefore, the reduced CPS-1 activity in pigs fed the CON or LP diets supplemented with 360 mg/kg NAM may indicate a decline in AA utilization for urea synthesis, enhancing nitrogen retention [18].

NAM supplementation in the CON and LP diets increased glucose levels and PDH activity in the serum, liver, and ileum. PDH plays a central role in glucose oxidation and glycogen metabolism, facilitating the TCA cycle [31]. The enhanced PDH activity observed with the 360 mg/kg NAM diet supports the functionality of the TCA cycle in nutrient metabolism. Additionally, NAM supplementation increased the ileal activity of CS and GDH, which are the key enzymes in the TCA cycle [32]. CS initiates the TCA cycle, enabling the entry of acetyl-CoA from pyruvate, where it condenses with oxaloacetate to generate citrate [33]. GDH is a glutaminolysis enzyme that converts glutamate into α-ketoglutarate, which enters the TCA cycle [34]. These findings suggest that NAM promotes anaplerotic AA recycling to sustain TCA cycle intermediates [35].

Although dietary CP levels did not significantly influence *mTORC1* expression, supplementing the LP diet with NAM enhanced *mTORC1* gene expression in the ileum. Proteins are hydrolyzed into AA and small peptides that are efficiently absorbed by the mucosa of the small intestine [36,37]. *mTORC1* is essential for maintaining intestinal mucosal integrity and regulating protein synthesis and nutrient metabolism in mammals. Notably, the villi-to-crypt ratio is a key indicator for evaluating intestinal structural integrity and nutrient absorption. Additionally, upregulated *GLUT1* and *GLUT2* expression along with *ASCT1* suggest that increased glucose and neutral AAs are delivered into the cell [38,39]. Pigs fed NAM-supplemented diets had an improved ileal villus length to crypt depth ratio, gene expression of glucose and AA transporters, and enhanced activity of enzymes involved in the TCA cycle in the ileum. Therefore, 360 mg/kg NAM supplementation improved intestinal morphological structure and promoted the absorption and utilization of AA.

The gut microbiota is a crucial player in maintaining host energy homeostasis [40]. The interplay between the gut microbiome and its metabolites can provide insights into mechanisms underlying energy metabolic disorders [41]. Additionally, promoting NAD^+^ expression can help mitigate host energy imbalances associated with dysregulated gut microbiota [42]. However, the relationship between dietary protein levels and gut microbiota composition remains contentious, with conflicting findings in the literature [43,44,45]. In this study, pigs fed the LP diet had a higher abundance of *Akkermansia muciniphila* (*A. muciniphila*) in their intestines than those fed the CON diet. *A. muciniphila* thrives on intestinal mucins and has a competitive advantage over other microorganisms under conditions of limited nutrition [46]. Interestingly, the administration of *A. muciniphila* in mouse models has been associated with reduced body and fat weight, likely due to diminished energy absorption in the gut [47]. Additionally, pigs receiving a diet supplemented with 360 mg/kg NAM exhibited a decreased relative abundance of *Lactobacillus* in the ileum, a core microbial genus in the porcine gastrointestinal tract [48]. Notably, the abundance of *Lactobacillus* has been inversely correlated with body weight [49]. These findings suggest that NAM supplementation modulates gut microbiota composition, particularly the abundance of *A. muciniphila* and *Lactobacillus* species, which may improve intestinal nutrient absorption and subsequently promote weight gain.

The intestine plays a pivotal role in nutrient absorption and metabolism, and its selective permeability ensures the efficient transport of nutrients [50]. Dietary protein quality significantly affects purine absorption, as proteins are metabolized into AAs [51]. Metabolic intermediates are produced during protein hydrolysis into AAs [52]. These intermediates are further broken down and converted into purines when acted upon by specific enzymes secreted by the intestinal flora [53,54]. Notably, LP diets supplemented with 360 mg/kg NAM upregulated nucleotide and purine metabolism in ileal microbiota, suggesting that NAM enhances the efficiency of these biochemical pathways. Nucleotide metabolism encompasses nucleotide synthesis, degradation, and interconversion, which are crucial for DNA, RNA, and high-energy molecules such as ATP and GTP [55]. However, purine metabolism involves both purine catabolism and synthesis, which are essential components of nucleotides such as adenosine triphosphate and guanosine triphosphate [56]. Enhanced nucleotide and purine metabolism increases the demand for precursor components, such as glycine, Gln, and aspartic acid, which are required for the synthesis of these molecules [57,58]. The limited use of AAs is attributed to improved nucleotide and purine metabolism, resulting in an effective allocation of AAs for nucleotide and purine synthesis rather than catabolism for energy generation [59]. Therefore, adding 360 mg/kg NAM into the LP diet increased AA use and reduced AA consumption, which may ensure that AAs were available for crucial intracellular functions.

Pigs in the LP + NAM group had a reduced abundance of *Lactobacillus* in their ileal microbiota. This finding aligns with previous research showing an inverse relationship between *Lactobacillus* abundance and purine absorption, as a higher presence of this genus is associated with reduced purine availability [60,61]. This result is also supported by correlation analysis results, which show that guanosine and inosine concentrations in the ileal microbiota of pigs in the LP + NAM group and the abundance of *Lactobacillus* were significantly negatively correlated compared to those in the LP group. This finding suggests that *Lactobacillus* plays a pivotal role in mediating the alleviating effects of NAM on growth restrictions in pigs fed LP diets.

## 5. Conclusions

In conclusion, adding 360 mg/kg NAM to LP diets decreased hepatic urea synthesis, improved intestinal morphology, enhanced intestinal glucose and AA absorption, and upregulated the activities of TCA cycle enzymes, such as PDH, CS, and GDH1, in the ileum, without toxicity to growing–finishing pigs. It also improved nucleotide and purine metabolism in intestinal microorganisms. Nicotinamide-mediated modulation of intestinal microbiota metabolism appears to enhance porcine gastrointestinal homeostasis and nutrient utilization efficiency, consequently reducing nitrogen excretion while optimizing growth performance in swine production systems.

## Figures and Tables

**Table 1:** Real-time fluorescent quantitative PCR primers.

Gene	Primers	Sequences (5′ to 3′)	Annealing Temperature (°C)	Accession No.
*NAMPT*	F	CCGACTCGTACAAGGTTACTC	58	NM_001031793.2
	R	TGGATCTTCTCTTTGGTCACTAC	58	
*SIRT1*	F	GCTATTGGGTACCGAGATAACCTT	60	NM_001145750.2
	R	TCGAGGATCTGTGCCAATCA	59	
*LKB1*	F	TGGGGTCACGCTCTACAAC	59	NM_001407255.1
	R	CTGCCGGATCTGCTGTATGG	60	
*AMPK-1*	F	GGCAAAGTGAAGGTTGGCA	59	NM_001167633.1
	R	AGATGGTGTACTGATGACCTGG	59	
*PPARα*	F	GAAGGTTGCAAGGGCTTCTT	59	NM_001044526.1
	R	TGGCTTTTTCAGACCTTGGC	59	
*PGC-1α*	F	CCCACAGAGACCCGAAACAG	60	NM_213963.2
	R	ACCCTTGGGGTCATTTGGTG	60	
*ACC1*	F	GGATGAACCGTCTCCCTTGG	58	NM_001114269.1
	R	CCAGACATGCTGGACCTCAT	57	
*mTORC1*	F	GTGAAACCGGAGGCCCTAAA	60	XM_003127584.6
	R	CAGAAAGGACACCAGCCGAT	60	
*FOXO1*	F	CTGAGTGAGTGAGCAGGCTA	59	NM_214014.3
	R	GGAAAAGTGTCTTCGCTGCC	60	
*CREB*	F	GGAGCTTGTACCACCGGTAA	59	NM_001361427.1
	R	CGGTGGGAGCAGATGATGTT	60	
*GLUT1*	F	CGCTTCCTGCTCATCAACC	59	XM_021096908.1
	R	GACCTTCTTCTCCCGCATC	58	
*GLUT2*	F	TCTTTGGTGGGATGCTTGGA	59	NM_001097417.1
	R	AAGCCTGAAATTAGCCCACAG	59	
*ASCT1*	F	GTGACCCACAACACGAGCAA	61	XM_021087450.1
	R	TGCAAATGGCGTGACGAG	59	
*ASCT2*	F	CAAGATTGTGGAGATGGAGGAT	58	XM_003355984.4
	R	TTGCGAGTGAAGAGGAAGTAGAT	59	
*SLC7A5*	F	TCAACCCCTACAGAAACCTGC	60	XM_047790216.1
	R	GACAGGGTGGTGAAGTAGGC	60	
*GDH1*	F	AGGGCTTTATTGGTCCTGGC	60	NM_001244501.2
	R	TCCACGACCAGTAGCAGAGA	60	
*NMNAT1*	F	GGCCAGTAGCGTGAGTTACA	60	XM_021095304.1
	R	AAAAGGAAACCTCCGACCCC	60	
*NADSYN1*	F	GGAATCTCCGGTCACTCAGG	60	XM_021082640.1
	R	ACTCCTCTGTTTGCCGACTC	60	
*NMRK1*	F	GAGGGTTAGTGAGAGGCGTG	60	XM_003121961.4
	R	ACCATTTGTCACACCACCGA	60	
*β-actin*	F	TTCTAGGCGGACTTGCAGC	60	XM_021086047.1
	R	GCTTCTCAGCAGACAGGAGG	60	

Abbreviations: *NAMPT*, nicotinamide phosphoribosyltransferase; *SIRT1*, sirtuin 1; *LKB1*, liver kinase B1; *AMPK-1*, AMP-activated protein kinase 1; *PPARα*, peroxisome proliferator-activated receptor-α; *PGC-1α*, peroxisome proliferator-activated receptor gamma coactivator 1 alpha; *ACC1*, acetyl-CoA carboxylase 1; *mTORC1*, mammalian target of rapamycin 1; *FOXO1*, forkhead box transcription factor O1; *CREB*, cAMP response element-binding protein; *GLUT1*, glucose transporter 1; *GLUT2*, glucose transporter 2; *ASCT1*, alanine-serine-cysteine transporter 1; *ASCT2*, alanine-serine-cysteine transporter 2; *SLC7A5*, solute carrier family 7 member 5; *GDH1*, glutamine dehydrogenase 1; *NMNAT1*, NMN adenylyl transferase 1; *NADSYN1*, NAD synthase 1; *NMRK1*, nicotinamide ribonucleoside kinase 1.

**Table 2:** Effects of dietary CP and niacinamide on serum metabolites and hormone levels in growing–finishing pigs.

Items	Normal-Protein Diet		Low-Protein Diet		SEM	*p*-Value		
	30 mg/kg NAM	360 mg/kg NAM	30 mg/kg NAM	360 mg/kg NAM		CP	NAM	CP × NAM
BUN (mmol/L)	7.2 ^a^	6.3 ^ab^	5.9 ^b^	6.2 ^ab^	0.26	0.012	0.266	0.023
GLU (mmol/L)	3.6	5.9	4.5	6.9	0.69	0.161	0.003	0.962
TG (mmol/L)	0.52	0.50	0.54	0.67	0.06	0.128	0.406	0.225
HDL-C (mmol/L)	1.2 ^b^	1.7 ^a^	1.5 ^ab^	1.2 ^b^	0.10	0.432	0.276	0.002
LDL-C (mmol/L)	2.4	2.4	2.3	2.8	0.15	0.458	0.175	0.175
GH (ng/mL)	2.6 ^a^	2.1 ^ab^	1.3 ^b^	1.6 ^b^	0.23	0.001	0.525	0.010
FINS (mIU/L)	1.7	2.3	1.6	1.4	0.32	0.123	0.575	0.193
GC (pg/mL)	12	18	12	17	3.8	0.840	0.204	0.953
GPT (U/L)	24	22	28	30	3.6	0.148	0.998	0.627
GOT (U/L)	9.6	7.6	5.7	14	3.7	0.748	0.409	0.179
PDH (ng/L)	1.8	3.7	1.3	2.7	0.34	0.042	<0.001	0.487
LDH (U/L)	415	414	467	363	53	0.990	0.327	0.340
HORA-IR	0.26	0.55	0.32	0.42	0.06	0.576	0.005	0.122

Abbreviations: BUN, blood urea nitrogen; GLU, glucose; TG, triglyceride; HDL-C, high-density lipoprotein cholesterol; LDL-C, low-density lipoprotein cholesterol; GH, growth hormone; FINS, fasting insulin; GC, glycogen; GPT, glutamic-pyruvic transaminase; GOT, glutamic-oxaloacetic transaminase; PDH, pyruvate dehydrogenase; LDH, lactate dehydrogenase; HOMA-IR, homeostasis model assessment of insulin resistance. ^a,b^ Values within a row with different superscripts differ significantly (*p* < 0.05). Data are shown as the mean ± SEM (*n* = 6).

**Table 3:** Effects of dietary CP and niacinamide on liver urea cycle metabolic enzyme activity in growing–finishing pigs.

Items	Normal-Protein Diet		Low-Protein Diet		SEM	*p*-Value		
	30 mg/kg NAM	360 mg/kg NAM	30 mg/kg NAM	360 mg/kg NAM		CP	NAM	CP × NAM
TP (mg/g)	94	110	94	105	5.5	0.957	0.107	0.512
GPT (U/g)	14	12	10	13	2.1	0.493	0.875	0.260
GOT (U/g)	64	59	64	58	4.9	0.916	0.262	0.911
CPS-1 (nmol/min/mg prot)	18	13	17	15	1.3	0.900	0.013	0.254
OTC (nmol/min/mg prot)	26	27	27	27	0.44	0.149	0.271	0.177
GS (μmol/h/mg prot)	207	189	170	154	16	0.034	0.947	0.300
GLS (U/mg prot)	17	11	10	10	2.2	0.093	0.236	0.228

TP, total protein; GPT, glutamic-pyruvic transaminase; GOT, glutamic-oxaloacetic transaminase; CPS-1, carbamyl phosphate synthetase-I; OTC, ornithine transcarbamylase; GS, glutamine synthetase; GLS, glutaminase. Data are shown as the mean ± SEM (*n* = 6).

**Table 4:** Effects of dietary CP and niacinamide on the liver and ileum activities of TCA cycle enzymes in growing–finishing pigs.

Items	Standard-Protein Diet		Low-Protein Diet		SEM	*p*-Value		
	30 mg/kg NAM	360 mg/kg NAM	30 mg/kg NAM	360 mg/kg NAM		CP	NAM	CP × NAM
Liver tissue								
CS (U/mg prot)	11	11	9.4	12	1.39	0.934	0.420	0.270
GDH1 (U/mg prot)	9.8	9.8	6.4	8.5	0.90	0.017	0.265	0.234
CPT1 (U/mg prot)	41	38	44	37	2.04	0.597	0.035	0.416
PDH (U/mg prot)	6.7	14	5.6	14	1.51	0.630	<0.001	0.800
LDH (U/g prot)	2012	1936	2239	1653	250	0.912	0.322	0.204
HK (nmol/min/mg prot)	11	16	16	19	1.74	0.046	0.030	0.604
Ileum tissue								
CS (U/mg prot)	5.0 ^b^	11 ^a^	1.3 ^c^	2.4 ^bc^	0.82	<0.001	<0.001	0.008
GDH1 (U/mg prot)	10	14	5.6	7.5	1.0	<0.001	0.018	0.486
CPT1 (U/mg prot)	42	44	53	53	3.55	0.010	0.803	0.846
PDH (U/mg prot)	10	17	6.1	13	1.65	0.018	0.001	0.971
LDH (U/g prot)	2925	2382	3641	2577	448	0.325	0.092	0.570
HK (nmol/min/mg prot)	17	16	19	28	6.52	0.311	0.602	0.465

Abbreviations: CS, citrate synthase; GDH1, glutamate dehydrogenase 1; CPT1, carnitine palmitoyltransferase 1; PDH, pyruvate dehydrogenase; LDH, lactate dehydrogenase; HK, hexokinase. ^a–c^ Values within a row with different superscripts differ significantly (*p* < 0.05). Data are shown as the mean ± SEM (*n* = 6).

## Data Availability

Data will be made available on request.

## Supplementary Materials

The following supporting information can be downloaded at: https://www.mdpi.com/article/10.3390/ani15142088/s1, Table S1: Ingredients and composition of diets of barrows with body weights ranging from 35 to 65 kg in nitrogen balance trial 2 and growth performance trial 1 (DM basis, %); Table S2: Ingredients and composition of diets for pigs with body weights ranging from 65 to 100 kg in growth performance trial 1 (DM basis, %); Table S3: Differential metabolites in the ileal microbes of growing and fattening pigs; Table S4: The correlation between the metabolites with significant differences in the ileum digesta and the *Lactobacillus* and *Akkermansia* in the ileum microbiota; Figure S1, Sample dilution curve, species accumulation box plot, and principal coordinates analysis; Figure S2: Microbial metabolism in the ileum of growing and fattening pigs.

## Abbreviations

The following abbreviations are used in this manuscript:

NAMPT	nicotinamide phosphoribosyltransferase
SIRT1	sirtuin 1
LKB1	liver Kinase B1
AMPK-1	AMP-activated protein kinase 1
PPARα	peroxisome proliferator-activated receptor-α
PGC-1α	peroxisome proliferator-activated receptor gamma coactivator 1 alpha
ACC1	acetyl-CoA carboxylase 1
mTORC1	mammalian target of rapamycin 1
FOXO1	forkhead box transcription factor O1
CREB	cAMP response element-binding protein
GLUT1	glucose transporter 1
GLUT2	glucose transporter 2
ASCT1	alanine-serine-cysteine transporter 1
ASCT2	alanine-serine-cysteine transporter 2
SLC7A5	solute carrier family 7 member 5
GDH1	glutamine dehydrogenase 1
NMNAT1	NMN adenylyl transferase 1
NMRK1	nicotinamide ribonucleoside kinase 1
BUN	blood urea nitrogen
GLU	glucose
TG	triglyceride
HDL-C	high-density lipoprotein cholesterol
LDL-C	low-density lipoprotein cholesterol
GH	growth hormone
FINS	fasting insulin
GC	glycogen
GPT	glutamic-pyruvic transaminase
GOT	glutamic-oxaloacetic transaminase
PDH	pyruvate dehydrogenase
LDH	lactate dehydrogenase
HOMA-IR	homeostasis model assessment of insulin resistance
TP	total protein
UN	urea nitrogen
CPS-1	carbamyl phosphate synthetase-I
OTC	ornithine transcarbamylase
GS	glutamine synthetase
GLS	glutaminase
CS	citrate synthase
GDH	glutamate dehydrogenase
CPT1	carnitine palmitoyltransferase 1
HK	hexokinase
NAM	nicotinamide
LP	low-protein
CP	crude protein
NAD+	nicotinamide adenine dinucleotide
CON	control
TCA	tricarboxylic acid
