# Supplementary material for: Impacts of 360 mg/kg Niacinamide Supplementation in Low-Protein Diets on Energy and Nitrogen Metabolism and Intestinal Microbiota in Growing–Finishing Pigs
Source: Animals (Basel). 2025 Jul 15;15(14):2088. doi: 10.3390/ani15142088 (PMC12291630; doi:10.3390/ani15142088)
Supplement: Supplementary file 1 [file animals-15-02088-s001.zip › animals-3669607-supplementary.pdf]

Title: Impacts of 360 mg/kg Niacinamide Supplementation in Low-Protein Diets on Energy and Nitrogen Metabolism and Intestinal Microbiota in Growing–Finishing Pigs

Authors: Xiaoyi Long[1], Haiyang Wei[1], Zhenyang Wang[1], Zhiru Tang[1], Yetong Xu[1], Xie Peng[1], Liuting Wu[1,2,*] and Zhihong Sun[1,*]

Affiliations:

[1]Research Center for Bio-feed and Molecular Nutrition, College of Animal Science and Technology, Southwest University, Chongqing 400715, China

[2]State Key Laboratory of Swine and Poultry Breeding Industry, College of Animal Science and Technology, Sichuan Agricultural University, Chengdu 611130, China

[*]Corresponding author: s765210@swu.edu.cn (Z.S.); 80295@sicau.edu.cn (L.W.)

Table S1

Ingredients and composition of diets of barrows with body weights ranging from 35 to 65 kg in nitrogen balance trial 2 and growth performance trial 1 (DM basis, %)

	Treatments			
	CON	CON + NAM	LP	LP + NAM
Ingredients				
Corn	70.67	70.64	77.56	77.53
Soybean meal	21.40	21.40	13.00	13.00
Wheat bran	3.12	3.12	3.64	3.64
Soybean oil	1.20	1.20	1.60	1.60
L-lysine HCl (98.5%)	0.54	0.54	0.74	0.74
DL-methionine (99%)	0.10	0.10	0.16	0.16
L-threonine (98.5%)	0.20	0.20	0.33	0.33
L-tryptophan (98%)	0.02	0.02	0.07	0.07
$CaHPO_4 \cdot 2H_2O$	0.70	0.70	0.80	0.80
$CaCO_3$	0.80	0.80	0.85	0.85
NaCl	0.25	0.25	0.25	0.25
NAM	0.003	0.036	0.003	0.036
Premix [1]	1.00	1.00	1.00	1.00
Total	100	100	100	100
Composition				
ME, MJ/kg[2]	13.8	13.8	13.8	13.8
CP[3]	15.9	15.9	13.1	13.1
Calcium[3]	0.60	0.60	0.60	0.60
Phosphorus[3]	0.50	0.50	0.50	0.50
Starch[3]	45.0	45.0	48.6	48.6
NDF[3]	10.7	10.7	10.4	10.4
ADF[3]	4.70	4.70	4.18	4.18
SID lysine[4]	1.05	1.05	1.05	1.05
SID methionine[4]	0.36	0.36	0.36	0.36
SID threonine[4]	0.64	0.64	0.64	0.64
SID tryptophan[4]	0.19	0.19	0.19	0.19
SID valline[4]	0.64	0.64	0.54	0.54
SID isoleucine[4]	0.55	0.55	0.46	0.46
SID leucine[4]	1.22	1.22	1.02	1.02
SID phenylalanine[4]	0.70	0.70	0.59	0.59
SID histidine[4]	0.41	0.41	0.35	0.35
SID arginine[4]	0.81	0.81	0.69	0.69
SID alanine[4]	0.79	0.79	0.67	0.67
SID asx (aspartate + asparagine)[4]	1.44	1.44	1.23	1.23
SID cystine[4]	0.23	0.23	0.20	0.20
SID glx (glutamate + Glutamine)[4]	2.58	2.58	2.19	2.19
SID glycine[4]	0.61	0.61	0.52	0.52
SID proline[4]	0.84	0.84	0.69	0.69
SID serine[4]	0.73	0.73	0.60	0.60
SID tyrosine[4]	0.52	0.52	0.45	0.45

Abbreviations: CON, basal diet + 30 mg/kg NAM; CON + NAM, basal diet + 360 mg/kg NAM; LP, low-protein diet + 30 mg/kg NAM; LP + NAM, low-protein diet + 360 mg/kg NAM; ME, metabolizable energy; NAM, nicotinamide; SID, standardized ileal digestibility.

[1] Providing the following per kg diet: Cu (as copper sulfate), 100 mg; Fe (as ferrous sulfate), 100 mg; Zn (as zinc oxide), 120 mg; Mn (as manganese sulfate), 20 mg; I (as calcium iodate), 0.3 mg; and Se (as sodium selenite), 0.3 mg; vitamin A, 3, 800 IU; vitamin D3, 800 IU; vitamin E, 10 IU; vitamin K, 1 mg; choline, 200 mg; pantothenic, 5 mg; vitamin B2, 2 mg; folic acid, 0.8 mg; vitamin B1, 1 mg; vitamin B6, 1 mg; biotin, 0.08 mg; vitamin B12, 0.01 mg.

[2] Calculated values.

[3] Analyzed values.

[4] Values for the concentrations of SID AAs in the diets were calculated using standardized ileal digestible coefficients for various ingredients provided by the NRC (2012).

Table S2

Ingredients and composition of diets for pigs with body weights ranging from 65 to 100 kg in growth performance trial 1 (DM basis, %)

	Treatments			
	CON	CON + NAM	LP	LP + NAM
Ingredients				
Corn	76.03	76.00	83.68	83.65
Soybean meal	15.62	15.62	7.20	7.20
Wheat bran	3.62	3.62	3.62	3.62
Soybean oil	1.20	1.20	1.40	1.40
L-lysine HCl (98.5%)	0.42	0.42	0.68	0.68
DL-methionine (99%)	0.11	0.11	0.15	0.15
L-threonine (98.5%)	0.08	0.08	0.22	0.22
L-tryptophan (98%)	0.02	0.02	0.07	0.07
$CaHPO_4 \cdot 2H_2O$	0.80	0.80	0.84	0.84
$CaCO_3$	0.85	0.85	0.89	0.89
NaCl	0.25	0.25	0.25	0.25
NAM	0.003	0.036	0.003	0.036
Premix [1]	1.00	1.00	1.00	1.00
Total	100	100	100	100
Composition				
ME, MJ/kg[2]	13.8	13.8	13.8	13.8
CP[3]	13.8	13.8	11.1	11.1
Calcium[3]	0.60	0.60	0.60	0.60
Phosphorus[3]	0.50	0.50	0.50	0.50
Starch[3]	49.2	49.2	52.8	52.8
NDF[3]	10.6	10.6	10.2	10.2
ADF[3]	4.38	4.38	3.82	3.82
SID lysine[4]	0.94	0.94	0.94	0.94
SID methionine[4]	0.34	0.34	0.34	0.34
SID threonine[4]	0.60	0.60	0.60	0.60
SID tryptophan[4]	0.18	0.18	0.18	0.18
SID valline[4]	0.57	0.57	0.46	0.46
SID isoleucine[4]	0.48	0.48	0.38	0.38
SID leucine[4]	1.08	1.08	0.87	0.87
SID phenylalanine[4]	0.62	0.62	0.47	0.47
SID histidine[4]	0.35	0.35	0.28	0.28
SID arginine[4]	0.73	0.73	0.58	0.58
SID alanine[4]	0.71	0.70	0.55	0.55
SID asx (aspartate + asparagine)[4]	1.25	1.25	0.94	0.94
SID cystine[4]	0.21	0.21	0.17	0.17
SID glx (glutamate + Glutamine)[4]	2.33	2.33	1.85	1.85
SID glycine[4]	0.54	0.54	0.46	0.46
SID proline[4]	0.72	0.72	0.56	0.56
SID serine[4]	0.64	0.64	0.50	0.50
SID tyrosine[4]	0.45	0.45	0.38	0.38

Abbreviations: CON, basal diet + 30 mg/kg NAM; CON + NAM, basal diet + 360 mg/kg NAM; LP, low-protein diet + 30 mg/kg NAM; LP + NAM, low-protein diet + 360 mg/kg NAM; ME, metabolizable energy; NAM, nicotinamide; SID, standardized ileal digestibility.

[1] Providing the following per kg diet: Cu (as copper sulfate), 100 mg; Fe (as ferrous sulfate), 100 mg; Zn (as zinc oxide), 120 mg; Mn (as manganese sulfate), 20 mg; I (as calcium iodate), 0.3 mg; and Se (as sodium selenite), 0.3 mg; vitamin A, 3, 800 IU; vitamin D3, 800 IU; vitamin E, 10 IU; vitamin K, 1 mg; choline, 200 mg; pantothenic, 5 mg; vitamin B2, 2 mg; folic acid, 0.8 mg; vitamin B1, 1 mg; vitamin B6, 1 mg; biotin, 0.08 mg; vitamin B12, 0.01 mg.

[2] Calculated values.

[3] Analyzed values.

[4] Values for the concentrations of SID AAs in the diets were calculated using standardized ileal digestible coefficients for various ingredients provided by the NRC (2012).

Table S3

Differential metabolites in the ileal microbes of growing and fattening pigs

Name	VIP	P-value	Fold change	Regulated
CON vs CON + NAM				
L-Aspartate	1.2	0.03	0.38	down
Glutamine	1.4	0.02	0.42	down
Tyrosine	1.2	0.03	0.33	down
Threonine	1.4	0.01	0.34	down
L-Glutamic-acid	1.3	0.02	0.49	down
Serine	1.2	0.03	0.41	down
L-citrulline	1.1	0.07	0.48	down
L-Cystine	1.1	0.05	0.30	down
L-Asparagine	1.5	0.01	0.25	down
Ornithine	1.3	0.04	0.38	down
Guanosine-diphosphate	1.2	0.14	0.30	down
dTMP	1.5	0.03	0.22	down
dAMP	1.6	0.01	0.19	down
dCMP	1.5	0.03	0.21	up
Oxaloacetate	1.0	0.21	4.52	down
Glycerol-3-phosphate	1.2	0.06	0.42	down
CON vs LP				
Guanosine-diphosphate	1.6	0.10	0.26	down
dTMP	2.0	0.03	0.21	down
dAMP	2.3	0.00	0.17	down
dCMP	2.1	0.02	0.16	down
Citric-acid	1.1	0.07	2.04	up
Oxaloacetate	1.4	0.15	5.53	up
D(+)-Glucose	1.1	0.51	0.50	down
Trehalose-6-phosphate	1.0	0.41	0.15	down
LP vs LP + NAM				
Flavin-mononucleotide	1.3	0.54	2.0	up
Guanosine-diphosphate	1.3	0.55	2.0	up
dTMP	1.4	0.33	2.7	up
dAMP	1.4	0.29	3.0	up
dCMP	1.5	0.26	3.3	up
Guanosine	1.6	0.21	4.5	up
Adenine	1.6	0.32	0.4	down
Inosine	1.2	0.41	3.2	up
Isocitric-acid	1.3	0.23	0.5	down
Oxaloacetate	1.6	0.18	0.3	down
Pyruvic-acid	1.6	0.26	6.6	up
CON + NAM vs LP + NAM				
L-Aspartate	1.3	0.15	2.1	up
Tyrosine	1.3	0.15	2.4	up
Itaconic-acid	1.8	0.04	0.45	down
L-Cystine	1.2	0.20	2.3	up

Adenine	1.1	0.30	0.46	down
Isocitric-acid	1.6	0.16	0.13	down
Oxaloacetate	1.2	0.26	0.31	down
D(+)-Glucose	1.0	0.31	3.6	up

Abbreviations: CON, basal diet + 30 mg/kg NAM; CON + NAM, basal diet + 360 mg/kg NAM; LP, low-protein diet + 30 mg/kg NAM; LP + NAM, low-protein diet + 360 mg/kg NAM. n = 6.

Table S4

The correlation between the metabolites with significant differences in the ileum digesta and the *Lactobacillus* and *Akkermansia* in the ileum microbiota

Name	Bacterial	Correlation	*P*
CON vs CON + NAM			
L-Aspartate	*Lactobacillus*	0.40	0.22
Glutamine	*Lactobacillus*	0.52	0.10
Tyrosine	*Lactobacillus*	0.47	0.15
Threonine	*Lactobacillus*	0.49	0.13
L-Glutamic-acid	*Lactobacillus*	0.40	0.23
Serine	*Lactobacillus*	0.44	0.18
L-citrulline	*Lactobacillus*	0.54	0.09
L-Cystine	*Lactobacillus*	0.36	0.27
L-Asparagine	*Lactobacillus*	0.60	0.05
Ornithine	*Lactobacillus*	0.68	0.02
Flavin-mononucleotide	*Lactobacillus*	0.28	0.40
Guanosine-diphosphate	*Lactobacillus*	0.27	0.42
dTMP	*Lactobacillus*	0.34	0.31
dAMP	*Lactobacillus*	0.38	0.25
dCMP	*Lactobacillus*	0.38	0.25
Oxaloacetate	*Lactobacillus*	-0.02	0.95
Glycerol-3-phosphate	*Lactobacillus*	0.66	0.03
CON vs LP			
Phosphorylethanolamine	*Akkermansia*	-0.06	0.85
Guanosine-diphosphate	*Akkermansia*	0.20	0.54
dTMP	*Akkermansia*	-0.22	0.48
dAMP	*Akkermansia*	-0.35	0.27
dCMP	*Akkermansia*	-0.34	0.27
AMP	*Akkermansia*	-0.22	0.50
UDP-GlcNAc	*Akkermansia*	-0.22	0.50
ADP	*Akkermansia*	-0.22	0.50
Citric-acid	*Akkermansia*	0.58	0.05
Oxaloacetate	*Akkermansia*	-0.05	0.87
D(+)-Glucose	*Akkermansia*	0.38	0.23
D-Fructose-6-phosphate	*Akkermansia*	-0.22	0.50
D-Glucose-1-phosphate	*Akkermansia*	-0.22	0.50
D-Glucose-6-phosphate	*Akkermansia*	-0.22	0.50
Trehalose-6-phosphate	*Akkermansia*	0.08	0.81
Glyceraldehyde-3-phosphate	*Akkermansia*	-0.22	0.50
Dihydroxyacetone-phosphate	*Akkermansia*	-0.22	0.50
Phosphorylethanolamine	*Lactobacillus*	0.21	0.52
Guanosine-diphosphate	*Lactobacillus*	0.40	0.20
dTMP	*Lactobacillus*	0.45	0.14
dAMP	*Lactobacillus*	0.49	0.11
dCMP	*Lactobacillus*	0.49	0.11

AMP	*Lactobacillus*	0.07	0.83
UDP-GlcNAc	*Lactobacillus*	0.07	0.83
ADP	*Lactobacillus*	0.07	0.83
Citric-acid	*Lactobacillus*	-0.23	0.47
Oxaloacetate	*Lactobacillus*	-0.20	0.52
D(+)-Glucose	*Lactobacillus*	0.05	0.88
D-Fructose-6-phosphate	*Lactobacillus*	0.07	0.83
D-Glucose-1-phosphate	*Lactobacillus*	0.07	0.83
D-Glucose-6-phosphate	*Lactobacillus*	0.07	0.83
Trehalose-6-phosphate	*Lactobacillus*	-0.41	0.19
Glyceraldehyde-3-phosphate	*Lactobacillus*	0.07	0.83
Dihydroxyacetone-phosphate	*Lactobacillus*	0.07	0.83
CON vs LP + NAM			
L-Asparagine	*Lactobacillus*	0.52	0.09
Phosphorylethanolamine	*Lactobacillus*	0.32	0.31
Guanosine	*Lactobacillus*	-0.86	0.00
Adenine	*Lactobacillus*	0.31	0.33
Inosine	*Lactobacillus*	-0.87	0.00
D-Ribulose-5-phosphate	*Lactobacillus*	0.20	0.54
Trehalose-6-phosphate	*Lactobacillus*	0.32	0.31
Phosphoenolpyruvic-acid	*Lactobacillus*	0.74	0.01
LP vs LP + NAM			
Flavin-mononucleotide	*Akkermansia*	-0.07	0.82
Guanosine-diphosphate	*Akkermansia*	-0.07	0.82
dTMP	*Akkermansia*	-0.16	0.61
dAMP	*Akkermansia*	0.08	0.80
dCMP	*Akkermansia*	-0.25	0.44
AMP	*Akkermansia*	0.06	0.86
Guanosine	*Akkermansia*	-0.45	0.15
Adenine	*Akkermansia*	0.09	0.79
Inosine	*Akkermansia*	-0.45	0.14
ADP	*Akkermansia*	0.06	0.86
Isocitric-acid	*Akkermansia*	-0.12	0.70
Oxaloacetate	*Akkermansia*	0.31	0.33
Pyruvic-acid	*Akkermansia*	-0.42	0.18
Trehalose-6-phosphate	*Akkermansia*	0.26	0.41
Flavin-mononucleotide	*Lactobacillus*	-0.07	0.83
Guanosine-diphosphate	*Lactobacillus*	-0.07	0.83
dTMP	*Lactobacillus*	-0.03	0.93
dAMP	*Lactobacillus*	0.03	0.93
dCMP	*Lactobacillus*	-0.27	0.39
AMP	*Lactobacillus*	0.24	0.44
Guanosine	*Lactobacillus*	-0.73	0.01
Adenine	*Lactobacillus*	0.29	0.35
Inosine	*Lactobacillus*	-0.59	0.04
ADP	*Lactobacillus*	0.24	0.44
Isocitric-acid	*Lactobacillus*	0.11	0.74
Oxaloacetate	*Lactobacillus*	0.62	0.03
Pyruvic-acid	*Lactobacillus*	-0.01	0.97

Trehalose-6-phosphate	*Lactobacillus*	-0.29	0.35

Abbreviations: CON, basal diet + 30 mg/kg NAM; CON + NAM, basal diet + 360 mg/kg NAM; LP, low-protein diet + 30 mg/kg NAM; LP + NAM, low-protein diet + 360 mg/kg NAM. n = 6.

Supplementary Figures:

Supplementary Figure 1: Principal coordinates analysis is generated of the Unweighted Unifrac Distance based on OTU counts and explains the largest variance between all samples. The sample dilution curve and species accumulation box plots, an analysis that describes the increase in species diversity with increasing sample size, are a useful tool for investigating the species composition of a sample and predicting the abundance of species in a sample, and are widely used in biodiversity and community surveys for determining the adequacy of sample sizes and for estimating species richness (Fig. S1A and B). The principal coordinates analysis is used to reflect differences in the composition of sample communities (Fig. S1C). The distance matrix of the samples was constructed using Unifrac analysis on the R language platform. Points with the same shape belonged to the same group, and each point indicated a replicate within the group; the closer the distance between the two points, the less the difference between the two samples' microbial communities.

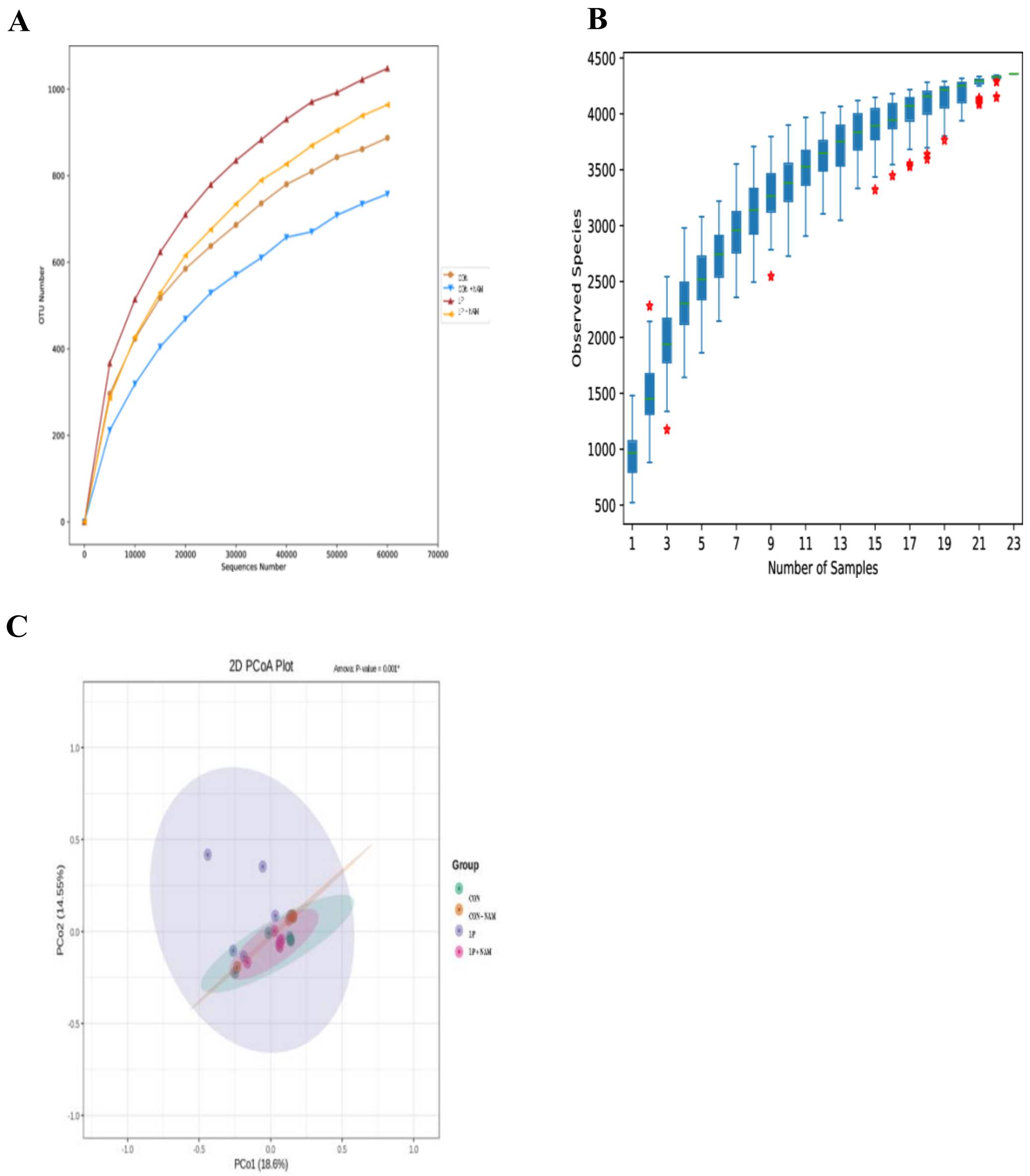

Figure S1. Sample dilution curve, species accumulation box plot, and principal coordinates analysis for ileal microbiota. (A) Sample dilution curve; (B) Species accumulation box plot (Significantly different domains were tested by Student's t test with Welch's correction); (C) Principal coordinates analysis.

Supplementary Figure 2: To find any variations in metabolites between the ileal digesta of growing and fattening pigs in each group, two-by-two comparisons were performed using principal component analysis (PCA) modeling (Fig. S2A-E). The different metabolites of each group were not entirely discernible on PCA, according to the PCA score plot. Subsequently, we performed two-by-two statistical analyses of samples from each group of ileal surimi of growing fattening pigs using the supervised multidimensional statistical method orthogonal partial least squares discriminant analysis (OPLS-DA) (Fig. S2F-J). In addition, this experiment also validated the above models by ranking to verify whether there was "overfitting" phenomenon, and the horizontal coordinate of the OPLS-DA ranking validation plot indicated the accuracy rate of the models, the vertical coordinate indicated the frequency of the accuracy rate of the 200 models in the 200 times of the permutation test, and R^2Y indicated the explanation rate of the models to the Y matrix, Q^2 represents the predictive ability of the model, theoretically, the closer the value of R^2 and Q^2 is to 1, the better the model is, and the lower it is, the worse the model's fitting accuracy is, usually, R^2 and Q^2 higher than 0.4 is acceptable. From the parameters of the OPLS-DA model for ileal digesta and the validation plot of the ranking, the differences between the groups and the different substances found were reliable,

and the R^2 and Q^2 were higher than 0.4 (Fig. S2K-O).

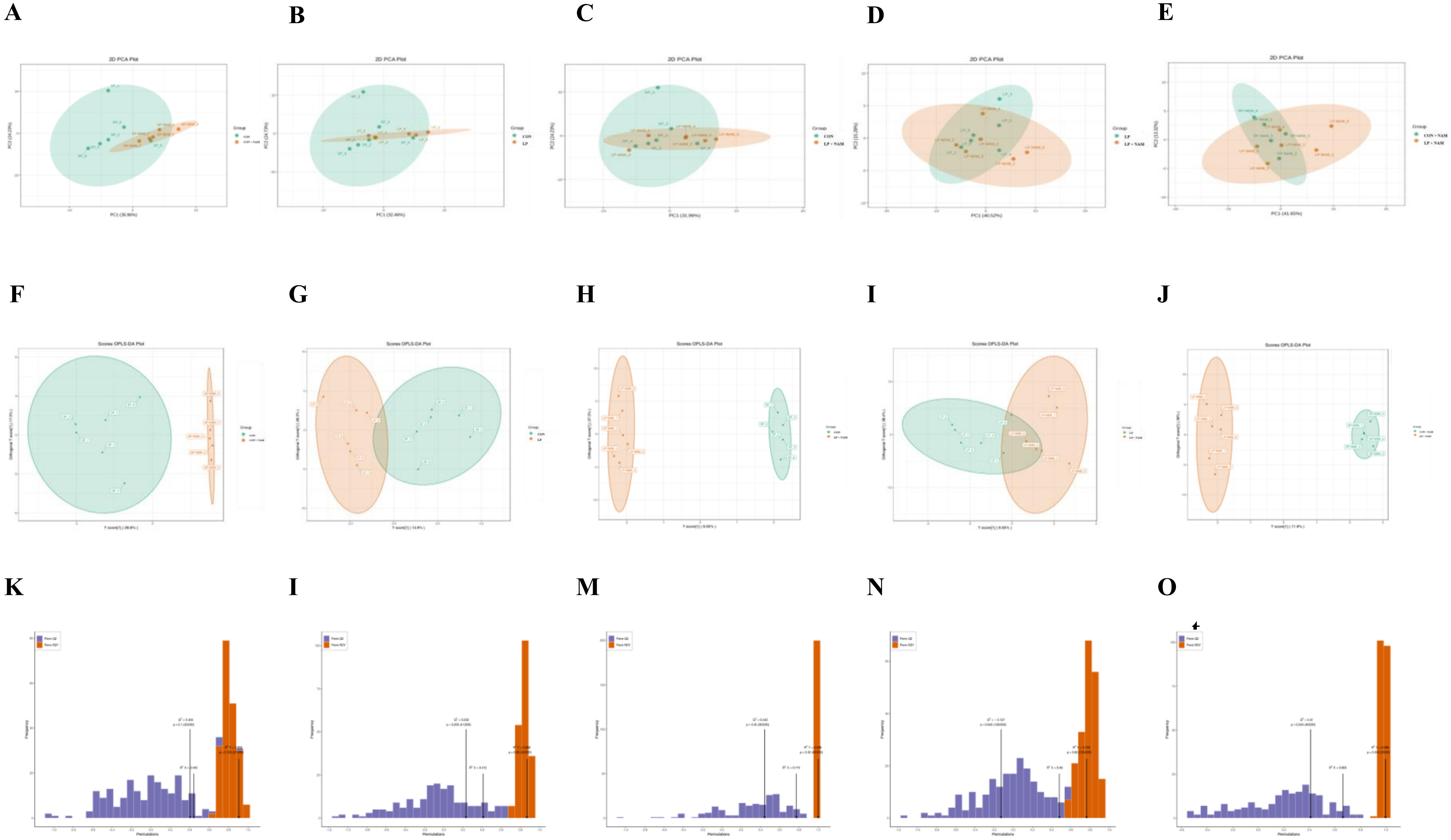
A
B
C
D
E
2D PCA Plot
F
G
H
I
J
Scores OPLS-DA Plot
K
I
M
N
O

Figure S2. Microbial metabolism in the ileum of growing and fattening pigs. (A–E) The PCA model. (F–J) The OPLS-DA score plots. (K–O) The OPLS-DA 200 permutation test. A, F, and K: CON vs CON + NAM groups; B, G, and I: CON vs LP groups; C, H, and M: CON vs LP + NAM groups; D, I, and N: LP vs LP + NAM groups; E, J, and O: CON + NAM vs LP + NAM groups.
